# Both High Reliability and Giant Electrocaloric Strength in BaTiO_3_ Ceramics

**DOI:** 10.1038/srep02895

**Published:** 2013-10-08

**Authors:** Yang Bai, Xi Han, Xiu-Cheng Zheng, Lijie Qiao

**Affiliations:** 1 Key Laboratory of Environmental Fracture (Ministry of Education), University of Science and Technology Beijing, Beijing 100083, China; 2 College of Chemistry and Molecular Engineering, Zhengzhou University, Zhengzhou 450001, China

## Abstract

BaTiO_3_ has a giant electrocaloric strength, |Δ*T*|/|Δ*E*|, because of a large latent heat and a sharp phase transition. The electrocaloric strength of a new single crystal, as giant as 0.48 K·cm/kV, is twice larger than the previous best result, but it remarkably decreased to 0.18 K·cm/kV after several times of thermal cycles accompanied by alternating electric fields, because the field-induced phase transition and domain switching resulted in numerous defects such as microcracks. The ceramics prepared from nano-sized powders showed a high electrocaloric strength of 0.14 K·cm/kV, comparable to the single crystals experienced electrocaloric cycles, because of its unique microstructure after proper sintering process. Moreover, its properties did not change under the combined effects of thermal cycles and alternating electric fields, i.e. it has both large electrocaloric effect and good reliability, which are desirable for practical applications.

Electrocaloric (EC) effect is a basic feature of ferroelectrics, referring to changes in entropy and temperature caused by electric field-induced variation of polarization states. In recent years, the attentions on EC effect grow rapidly, because the discovery of giant EC effects is promising to meet the needs in microelectronic and microelectromechanical devices for energy-efficient and environmental friendly solid-state refrigeration^1^. In 2006, a giant EC effect of *ΔT_max_* = 12 K, nearly an order of magnitude higher than before, was observed in PbZr_0.95_Ti_0.05_O_3_ thin films^2^, after that giant EC effects have been reported for various ferroelectric ceramic and polymer films^3^,^4^,^5^,^6^,^7^,^8^,^9^,^10^,^11^,^12^. Although the EC effect in thin films is greatly enhanced under ultrahigh fields of hundreds kV/cm, the EC strength |*ΔT*|/|*ΔE*| reduces at the same time, even lower than that of the bulk counterparts, including single crystal (SC) and ceramics^13^,^14^,^15^,^16^.

Recently, giant EC strengths were obtained in BaTiO_3_ SCs near the first order phase transitions (FOPT) in our previous work (0.24 K·cm/kV@5 kV/cm and 0.16 K·cm/kV@10 kV/cm)^13^,^14^ and in Moya's report (0.22 K·cm/kV@4 kV/cm)^15^. However, the low reliability of SC works against the practical applications. It was reported that cracks tend to nucleate and propagate in ferroelectric SCs because of the incompatible strain during the electric-field-induced (and/or temperature) domain switching^17^,^18^,^19^,^20^,^21^,^22^,^23^,^24^. This paper experimentally demonstrated the giant EC strength of BaTiO_3_ SCs and its rapid degradation with thermal cycles and alternating electric fields. More important, both high reliability and giant EC strength were achieved in BaTiO_3_ ceramics prepared from hydrothermal synthesized nano-sized powders, whose performances were comparable to the used SCs and far beyond the conventional ceramics.

## Results

Previous reports indicated that the EC effect near a FOPT is dominated by the energy change associated with the change of lattice structure, which can be two orders of magnitude higher than the entropy change of dipolar order^13^, while the sharpness of the transition is the key to a giant EC strength^15^. A perfect SC without defects behaves as an ideal crystal, where all lattice have the same energy barrier in phase transition and domain switching, so it will show a giant EC strength near FOPT.

**Figure 1:**
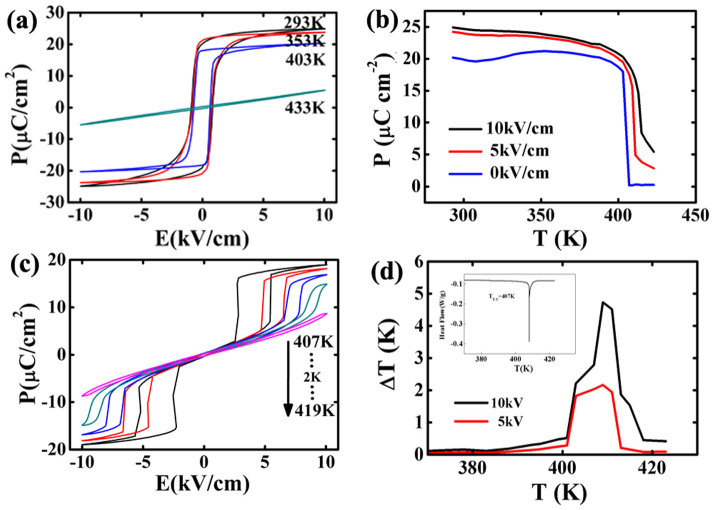
Ferroelectric and EC properties of the new SC. (a) *P*-*E* loops at different temperatures; (b) *P*-*T* curves under different electric fields; (c) double loops between 407 K and 413 K; (d) the temperature dependence of EC *ΔT*.

The *P*-*E* loops of a new BaTiO_3_ SC at different temperatures are shown in Fig. 1a. Besides an expected drop of polarization with increasing temperature (Fig. 1b), the normal hysteresis loop becomes double loop between 407 K and 413 K (Fig. 1c). The double hysteresis loop originates from a typical electric-field-induced phase transition (EFIPT) from paraelectric (P) to ferroelectric (F) phase^25^, not as that in aged BaTiO_3_ SCs assumed by Ren^26^. The double loop appears above T_1_ in Landau-Devonshire (LD) theory, i.e. the highest temperature where the low-temperature phase exists in a metastable state under zero-field. Here, T_1_ = 407 K is confirmed by both *P*-*E* loops and heat flow curves. The occurrence of double loop with regular shape indicates that there is little energy fluctuation in a new SC and the EFIPT carries out in all lattices uniformly. The good consistency of the phase transition is also reflected by the horizontal heat flow curve below T_1_ (inset in Fig. 1d). With increasing temperature, the critical field increases, indicating the ease of an EFIPT; while the width of one loop decreases, indicating the stability of the field-induced phase. This reflects the competition between the field-induced polarization order and the thermally excited disorder. The double loop disappears above 415 K, implying that external fields cannot induce phase transition any more, i.e. T_2_ in LD theory.

Based on the Maxwell relation, (∂P/∂T) = (∂S/∂E), the EC adiabatic temperature change *ΔT* for a material with density *ρ* and heat capacity *C* can be calculated by 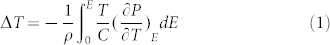
At 10 kV/cm field, *ΔT* reaches a maximum at 409 K, a bit higher than the phase transition temperature, and *ΔT*_max_ = 4.8 K is much higher than those in the previous reports, including that in our works (1.6 K@10 kV/cm)^14^ and those in Moya's reports (0.9 K@12 kV/cm and 0.87 K@4 kV/cm)^15^. In addition, the EC strength is as giant as |*ΔT*|/|*ΔE*| = 0.48 K·cm/kV, twice larger than the previous best result^1^,^2^,^3^,^4^,^5^,^6^,^7^,^8^,^9^,^10^,^11^,^12^,^13^,^14^,^15^,^16^,^27^.

**Figure 2:**
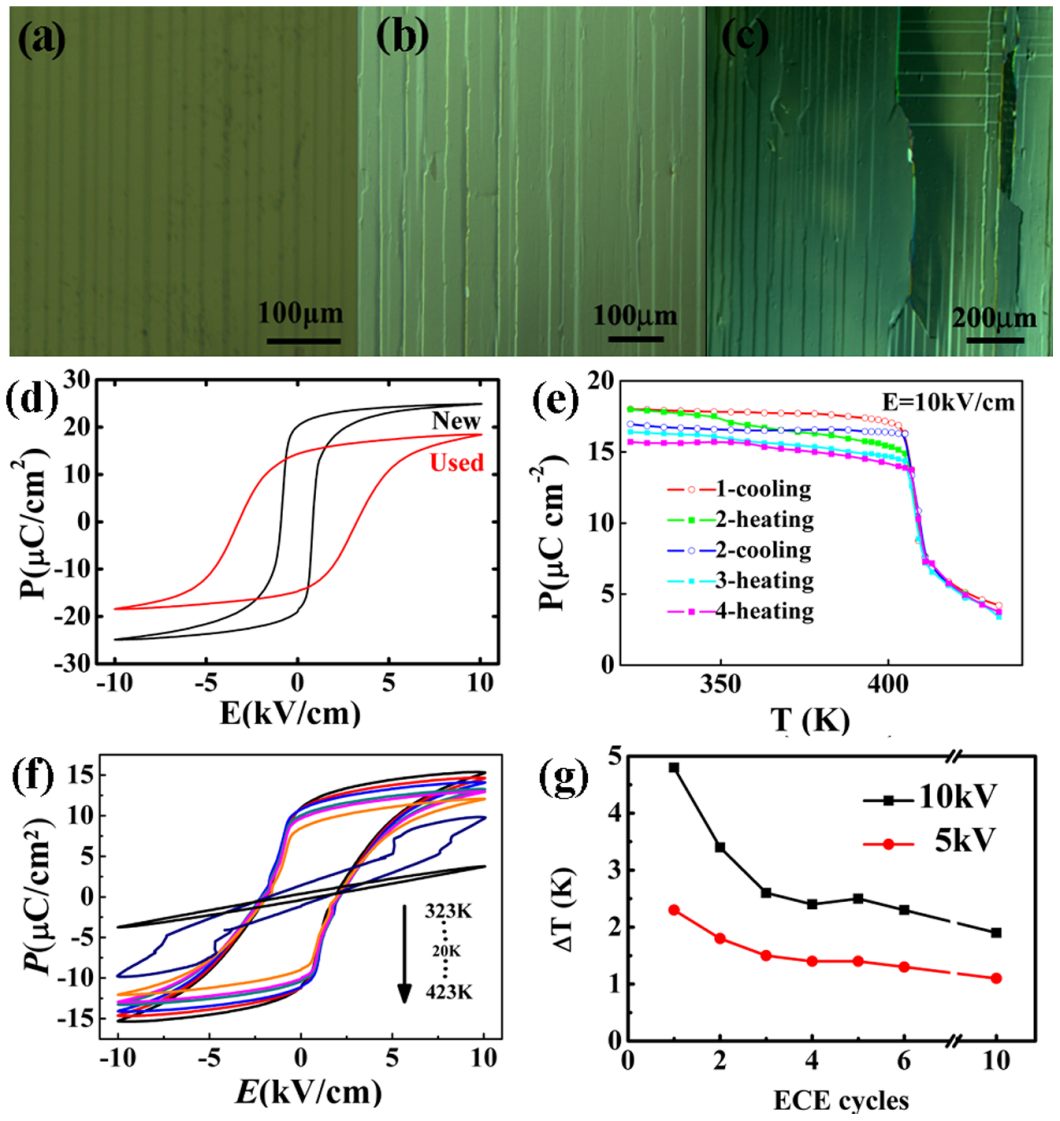
Ferroelectric and EC properties of the used SC, as well the evolution of microstructure with EC cycles. (a) *P*-*E* loops at different temperatures; (b) a comparison of room-temperature *P*-*E* loops between new and used SC; (c) *P*-*T* curves in a serial of EC cycles; (d) the variation of *ΔT_max_* with EC cycles; (e) the microstructure of the new SC before measurements, (f) after one EC cycle and (g) after five EC cycles.

However, the large amount of defects, such as microcracks, will occur in SC after several times of thermal cycles accompanied by alternating electric field cycles because of the incompatible strain during the lattice deformation^17^,^18^,^19^,^20^,^21^,^22^,^23^,^24^. (Hereafter, one EC cycle refers to a heating and cooling process from room temperature to 430 K, where three times of cycling electric fields were applied at certain temperatures.) As shown in Fig. 2a, there are alternately dark and bright domain stripes and no microcrack in a new SC. After several EC cycles, more and more microcracks nucleate parallel to domain strips and then propagate (Fig. 2b, 2c). The *P*-*E* loop becomes fatter and shorter (Fig. 2d), and *P* drops with increasing EC cycles (Fig. 2e), because the defects obstacle the domain switching and induce depolarization fields, and the cracks reduce the fields in the sample^28^,^29^,^30^. In addition, the energy fluctuation is enhanced and the sharpness of phase transition is weakened, therefore the double loop occurs more difficult in a smaller temperature range and the shape is irregular (Fig. 2f). As a result, Δ*T*_max_ drops dramatically to 1.8 K after several EC cycles (Fig. 2g), whose value is also confirmed by direct entropy change measurements either on heat capacity or on heat flow and is in agreement with the literature values^14^,^15^. It implies that new BaTiO_3_ SC has very outstanding EC performance but it is unreliable.

Although the degradation of EC performance in SC may be slowed down gradually (Fig. 2g), some irreversible effects appear, such as low fracture strength, easy breakdown and bad insulation. The bad insulation is reflected as the increase of apparent *P_r_* above the phase transition temperature.

**Figure 3:**
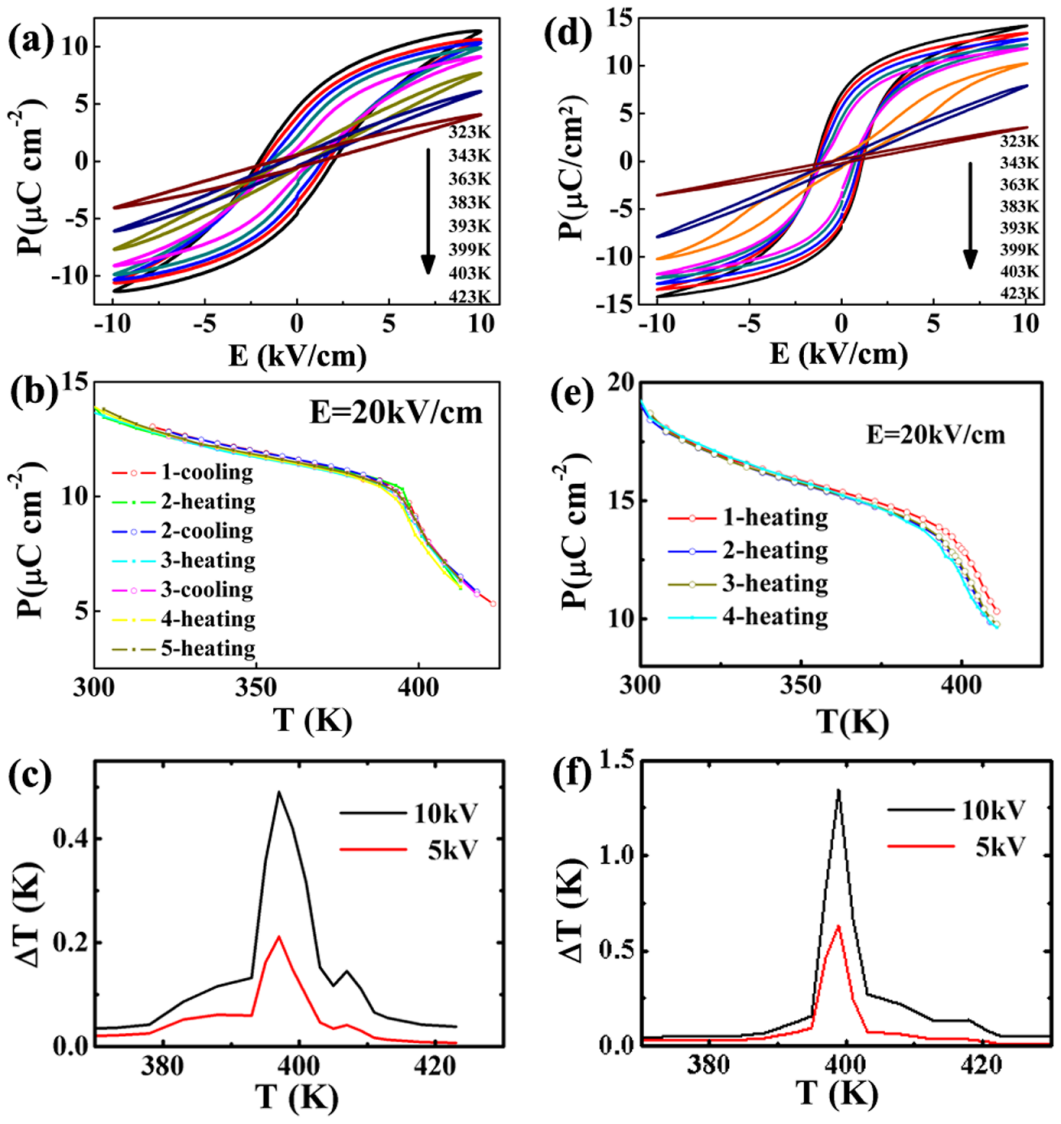
Ferroelectric and EC properties of M-Ceram (a, b, c) and N-Ceram (d, e, f). (a), (d) *P*-*E* loops at different temperatures; (b), (e) *P*-*T* curves in a serial of EC cycles; (c), (f) the temperature dependence of EC *ΔT*.

The polycrystalline ceramics have much better reliability than that of SCs because the grain boundaries buffer the stress during domain switching. The BaTiO_3_ ceramics also show typical ferroelectric hysteresis loops (Fig. 3a), but the polarization and EC effect keep steady after EC cycles (Fig. 3b), indicating good reliability. In addition, large amount of grain boundaries and pores are defects in the microstructures to reduce the polarization and enhance the energy fluctuation, so there is no double hysteresis loop (Fig. 3a) in the conventional ceramics (M-Ceram) prepared from ~1 μm starting powders by a solid-state reaction method, and the EC *ΔT*_max_ is as low as 0.5 K (Fig. 3c).

To retain good reliability and enhance EC effect, the microstructure of ceramics was modified by using hydrothermal synthesized powders with average size of ~35 nm (N-Ceram). The N-Ceram samples show both typical ferroelectric hysteresis loops (Fig. 3d) and high reliability (Fig. 3e) similar to those of M-Ceram. However, its EC effect, *ΔT*_max_ = 1.4 K, is much stronger than that of M-Ceram (Fig. 3f), which results from its unique microstructures.

**Figure 4:**
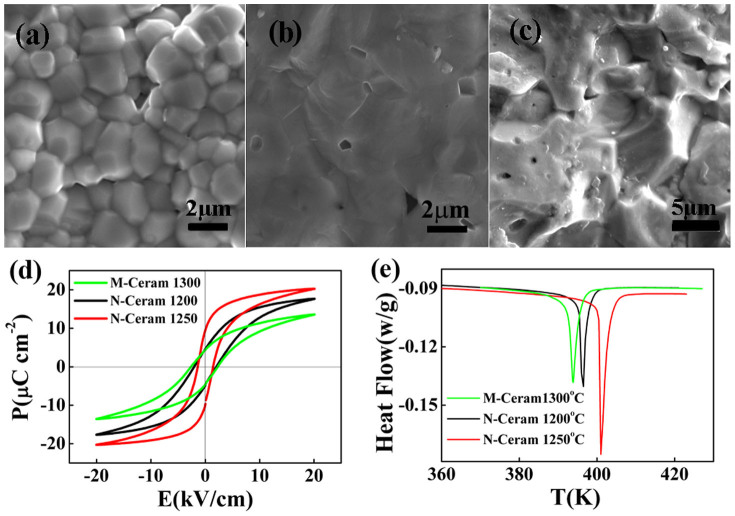
Comparison of microstructure and properties of M-Ceram and N-Ceram. (a) the microstructure of 1200°C sintered N-Ceram, (b) 1250°C sintered N-Ceram and (c) 1300°C sintered M-Ceram; (d) *P*-*E* loops; (e) heat flow curves.

The highly active nano-sized powders make the N-Ceram samples densified at a relatively low temperature of 1200°C (Fig. 4a). As the sintering temperature further rises to 1250°C, small grains integrate together and the boundaries become blurry (Fig. 4b). The blurry grain boundaries imply less lattice mismatch between neighboring grains, which are not found in the M-Ceram sample (Fig. 4c). The decrement of defects in the sintered ceramics reduces the obstacles for the domain switching, and lowers the energy fluctuation, so the *P*-*E* loops become more saturated (Fig. 4d), the endothermic peak becomes sharper (Fig. 4e), and the double *P*-*E* loop appears near the FOPT (Fig. 3d). Hence, N-Ceram sintered at 1250°C shows a large EC of *ΔT*_max_ = 1.4 K at 10 kV/cm field (Fig. 3f), i.e. a giant EC strength of 0.14 K·cm/kV. The EC strength is much higher than that of all conventional ceramics^1^,^2^,^3^,^4^,^5^,^6^,^7^,^8^,^9^,^10^,^11^,^12^,^13^,^14^,^15^,^16^ and relaxor SCs (such as PMN-PT)^27^, and is comparable to the used BaTiO_3_ SCs.

## Discussion

The heat low curves indicate that M-Ceram, N-Ceram and single crystals have similar latent heat about 0.90 ± 0.02 J/g, in agreement with literature^15^,^31^,^32^, but the sharpness of the endothermic peaks increases in turn (Fig. 4b). It indicates that the obvious differences in microstructure do not affect the total energy change of lattice structure at a FOPT, but influence the sharpness of the transition which determines the peak value of EC effects. Among all samples examined, N-Ceram shows both high EC effect and good reliability because its unique microstructures have fewer defects except for blurry grain boundaries.

## Methods

### Preparation of BaTiO_3_ samples

The (001) BaTiO_3_ single crystal was a commercially available product (Physcience Opto-Electronics Co., China). BaTiO_3_ ceramics were fabricated by conventional ceramics method, using hydrothermal synthesized nano-sized powders and solid-state reacted micron-sized powders, respectively.

In hydrothermal syntheses method, the HCl solution of 15.0% TiCl_3_ was added into the aqueous solution of BaCl_2_·2H_2_O based on an initial precursor molar ratio Ba/Ti of 1.6 and the pH was adjusted to 13.5 by adding 10 mol/L KOH solution. It was crystallized at 150°C for 8 h in an autoclave. After cooling down to room temperature, the pH was adjusted to 6.0. The final powders were obtained after filtration, wash and drying at 105°C. In solid-state reaction method, the raw materials of analytical reagent grade BaCO_3_, CaCO_3_, ZrO_2_ and TiO_2_ were mixed and calcined at 1000°C. Using the hydrothermal synthesized nano-sized powders or solid-state reacted micron-sized powders, the dry-pressed pellets were sintered at 1200–1350°C in air.

### Characterization of BaTiO_3_ single crystal and ceramics

The ferroelectric hysteresis loop was measured at 10 Hz using a TF2000 analyzer equipped with a temperature controller. The temperature dependence of polarization under certain electric field was extracted data from the upper branch of each loop, and then ∂*P*/∂*T* was obtained. Reversible adiabatic temperature change Δ*T* was calculated using Eq. (1).

The domain configuration in BaTiO_3_ single crystal was observed by polarized light microscopy, while the microstructure of ceramics was observed by scanning electron microscopy. The thermal characters of phase transition were measured within a temperature range of 100–150°C using a differential scanning calorimeter (DSC, TA Instruments Q2000).

## Author Contributions

Y.B. designed the experiments and analyzed the results. X.H. prepared the ceramics and characterized all samples. X.C.Z. prepared the nano powders. L.J.Q. guided the work and analysis. Y.B. wrote the paper.
